# Relationship between perceived social support and mental health among Chinese college football athletes: a moderated mediation model

**DOI:** 10.1186/s40359-023-01357-2

**Published:** 2023-10-11

**Authors:** Zongyu Liu, Xiuhan Zhao, Liangyu Zhao, Liguo Zhang

**Affiliations:** School of Physical Education, Shandong University, Jinan, 250061 China

**Keywords:** Perceived social support, Mental health, Football athletes, Hopelessness, Psychological pressure

## Abstract

Previous researches have confirmed that perceived social support has a profound effect on individuals’ mental health. However, the effects and potential mechanisms of perceived social support on mental health of college athletes are still largely unknown, especially during and after the COVID-19 pandemic. Thus, the aim of this study was to explore the relationship between perceived social support and mental health in college football athletes, and to evaluate whether hopelessness and psychological pressure affected this relationship. A sample of 672 Chinese college football athletes (37.9% girls; *M*_age_ = 20.43 years; *SD*_age_ = 1.68) were investigated with the Perceived Social Support Scale (PSSS), the Kessler Psychological Distress Scale (K10), the Beck Hopelessness Scale (BHS) and the Perceived Stress Scale (PSS). Surveys were voluntary and anonymous. The findings revealed that, after adjusting for demographic factors, hopelessness mediated the relationship between Chinese college football athletes’ perceived social support and their mental health. Furthermore, psychological pressure moderated the negative association between perceived social support and hopelessness, and the association was stronger for them with high-level psychological pressure. These results underline the need for focused strategies in the prevention and treatment of mental health issues among Chinese college football athletes.

## Introduction

According to the World Health Organization (WHO), the novel corona virus disease (COVID-19), which broke out in December 2019, has become a global public health emergency [1]. It is characterized by wide spread, sudden onset and strong infectiveness, and has seriously affected individuals’ physical and mental health [2]. When in self-isolation in a completely closed or semi-closed state, the public has little social contact, stays in one place for long periods of time and can only learn about the virus through the internet [3]. College athletes are a unique group of young people who must tackle the challenges of college courses while maintaining optimal physical fitness, which puts male and female college athletes at potential risk for a variety of mental health problems [4]. Moreover, they may pay more attention to all kinds of unexpected events since they are in the postponement period of psycho-social, facing the pressure of competition as well as the degree of socialization is constantly improving [5]. The negative impact of COVID-19 pandemic on the mental health of college athletes has also been studied in recent years [6–8]. For example, Garver et al. conducted a cross-sectional design to assess the impact of the COVID-19 crisis on the mental health of college athletes, and found that depression, anxiety and stress levels were significantly elevated among them during the pandemic [9]. At present, the Chinese government attaches great importance to the identification of young football talents, and the mental health level of football athletes is closely related to the results of the matches. Therefore, regulating and improving the mental health of them in long-term daily life and training will have a positive impact on improving the team’s match results.

Perceived social support is the satisfaction of being supported, respected and understood in society [5], which is a process of cognitive evaluation and used as a predictor of mental health. Compared with actual social support, perceived social support should be more of a cognitive phenomenon, indicating an individual’s understanding and perception of different social relationships, which can better reflect the utilization of actual social support and have a greater impact on the them [10]. It has been shown that perceived social support positively correlates with well-being and helps individuals cope with stressful events [11]. Besides, research shows that the mental health benefits of social support are strongest in studies of perceived social support, and low-level perceived social support was a significant predictor of psychological problems and physical illness [12, 13]. According to the main effect model of social support, perceived social support have a positive function on individual mental health and can help individuals improve their perceived self-coping ability, reduce the evaluation of the severity of problems and the adverse effects of stress [14, 15]. College athletes with high-level perceived social support have more positive expectations and psychological energy when facing psychological difficulties during and after the COVID-19 pandemic, thus promoting their mental health status [16, 17]. Therefore, we expect that perceived social support is positively related to mental health (H1).

Hopelessness refers to the psychological state in which individuals lose confidence in the future and lack of initiative, which is a negative emotion [18]. College athletes are under great pressure and more likely to have mental health problems compared with the age-matched general population under the impact of the COVID-19 epidemic [19]. Previous researches have demonstrated a significant relationship between mental health, perceived social support, and hopelessness [20–22]. Research has shown that a lack of hope is associated with various manifestations of mental illness, which increases stress and negative expectations for the future and is not conducive to healthy mental development [23]. In addition, the hopelessness caused by the COVID-19 pandemic, especially for people with preexisting mental health conditions (such as depression), can lead to suicidal thoughts and seriously impair an individual’s physical and mental health [24]. Available evidence suggests that hopelessness has a negative impact on an individual’s social life experience and mental health [25]. The relationship between perceived social support and hopelessness has mostly been studied in populations with cancer or other chronic diseases. Pehlivan et al. found that reduced social support is associated with increased feelings of hopelessness, and the perception that more social support from family may be enough to prevent the onset of hopelessness [26]. Research by Somasundaram et al. has also shown that perceived social support has a positive effect on emotional well-being and reducing feelings of hopelessness [27]. Perceived social support enables individuals to achieve their dreams by providing positive impact, a sense of predictability, stability in life situations and promoting well-being [28], however, lack of perceived social support may lead to feelings of hopelessness. In addition, the basic condition for the establishment of hope is to obtain the encouragement and support of others, and when individuals get the support, they will be motivated to voluntarily achieve the goal [29]. This will make the individual feel the hope of the future, and the sense of hopelessness is significantly reduced [30]. According to the self-determination theory [31], it can be inferred that the innate growth tendency of individuals depends on the social environment. When the environment does not provide adequate nutrition (e.g. lack of social support), negative internal processes such as hopelessness are increased. In addition, the sense of hopelessness will lead individuals to make wrong judgments about life without practical reasons, which will lead to negative cognitive evaluation of life conditions and reduce their mental health status [32]. Therefore, we speculate that a high degree of perceived social support may further improve the mental health of college athletes by reducing their hopelessness. Given the evidence, we hypothesize that hopelessness would mediate the association between perceived social support and mental health (H2).

Indeed, while perceived social support has a significant impact on hopelessness and mental health, there are undeniably individual differences in this effect. Therefore, it is necessary to examine whether the relationship between perceived social support and hopelessness is mediated by other factors. Psychological pressure can be divided into benign pressure and bad pressure and is defined as the emotional and physical reactions that individuals experience when the demands of adaptation exceed their coping resources [33], which is significant correlated with perceived social support and mental health [34, 35]. In this study, psychological pressure/stress describes the negative psychological thoughts and feelings associated with pain. Research reveals that perceived social support has a positive impact on reducing stress levels and improving quality of life [36], and can help a person minimize their perceived stress or adopt healthy behaviors to deal with stressors [12]. According to a review of the pertinent literature, psychological pressure is one of the numerous elements that contribute to hopelessness, and it may even moderate the relationship between perceived social support and hopelessness. According to the stress vulnerability hypothesis, positive factors will lose their protective effect in the face of high pressure. Based on this, it is thought that social support loses its protective effect on the positive emotions of college football athletes under high stress, which in turn may increase their feelings of hopelessness [37]. The theory of social support resources emphasizes that social support, as an external protection resource, can provide continuous psychological energy for individuals. However, if psychological stress plays a role after individuals receive sufficient social support, their personal resources may be consumed by the pressure, which may weaken the negative effect of understanding social support on hopelessness [38]. Moreover, according to social capital theory [39], the more social resources an individual has, the more it can help an individual cope with and adjust to life and physical changes. Therefore, we speculate that the amount of perceived social resources will be particularly important when the individual has a strong sense of psychological pressure, and the amount of perceived social support will have a stronger negative impact on hopelessness. Therefore, it was expected that a high-level psychological stress could strength adverse effects of perceived social support on hopelessness, which in turn could moderate the mediation effect of hopelessness in the relationship between perceived social support and mental health (H3).

**Fig. 1:**
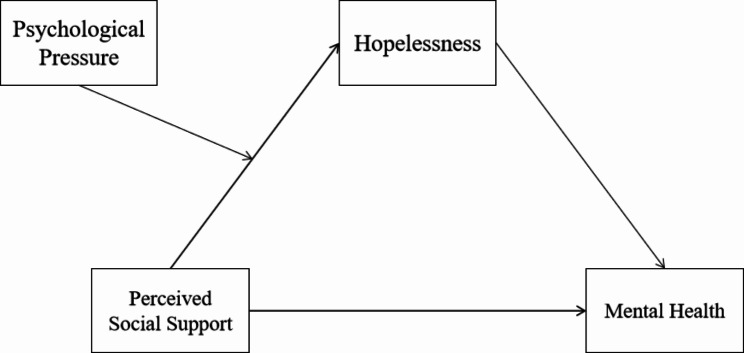
Structural framework

Based on the above literature, an integrated moderated mediation model was employed to investigated the following three questions (Fig. 1): (1) whether perceived social support would be positively related to mental health of Chinese college football athletes, (2) whether hopelessness mediate the link between perceived social support and mental health, and (3) whether the indirect association between perceived social support and mental health via hopelessness vary as a function of psychological pressure. This model focuses on the moderating and mediating mechanisms by which perceived social support affects college football athletes’ mental health in the Chinese context, which is an extension of the existing research. As the research progresses, it is no longer enough to explore the direct association between variables, and the introduction of mediating and moderating mechanisms can better reveal the process and conditions of the effect of perceived social support on improving college soccer players’ mental health, and provide a more in-depth explanation for the development of athletes’ mental health. The findings of this study may lead to evidence-based therapies for this demographic and be useful in improving college athletes’ mental health, boosting their physical performance, and help to identify national talent.

## Materials and methods

### Participants and procedures

The study was conducted during the COVID-19 pandemic in February 2022. A cross-sectional design was adopted to invite Chinese college football athletes to participate in the survey. 35 teams were randomly selected from the Chinese University Football League. In order to ensure the effectiveness and the high completion rate of the questionnaire, as well as the sense of responsibility of college athletes, we contacted the head coaches of various college sports teams. Coaches of each team sent an electronic questionnaire to all athletes via a link to the QR code through face-to-face meetings, and timely answered the confusion that college athletes encountered when filling out the questionnaire. Before completing the survey, all participants were informed of the purpose and procedure of the study and their right to withdraw from the study at any time. Before the survey was completed, each participant obtained informed consent online. A total of 694 college football athletes took part in the study. Participants who took less than 200 s to complete the questionnaire and those who did not complete the questionnaire were excluded from the sample. Based on these exclusion criteria, the final sample included 672 participants, with an effective recovery of 96.8%. There were 417 boys (62.1%) and 255 girls (37.9%), with an average age of 20.43 years and a standard deviation of 1.68 years. Participants ranged in age from 17 to 27. This study was approved by the Ethics Committee of Shandong University (No. 2021-1-114).

### Instruments

#### Demographic variables

Demographic characteristics were collected through several sociodemographic questions, including age, sex, urban-rural provenance, and duration of sports careers.

#### The perceived social support scale (PSSS)

The perceived Social support scale (PSS) consists of 12 items, which are divided into three dimensions: family support, friends support and other support. The scale was developed by Zimet et al. and has a favourable internal consistency (Cronbach’s α = 0.85–0.91) [40]. Each item is scored from 1 (strongly disagree) to 7 (strongly agree), and the total score ranges from 7 to 84. The higher the score is, the higher the level of social support the individual feels. In this study, the Cronbach’s α coefficients of this scale was 0.969.

#### The kessler psychological distress scale (K10)

The Kessler Psychological Distress Scale [41] consists of 10 self-rated items that measure the frequency of symptoms related to non-specific mental health conditions such as anxiety and stress levels experienced in the past 4 weeks. Each item has five answers assigned a score: almost none (1), occasionally (2), sometimes (3), most of the time (4), all of the time (5). The overall score ranges from 10 to 50, with higher scores indicating poorer mental health. In current study, all questions were scored in reverse. In this study, the Cronbach’s α coefficients of this scale was 0.916.

#### The beck hopelessness scale (BHS)

The Beck Hopelessness Scale (BHS) is a 20-item self-reported measure of hopelessness in the past 2 weeks. Example projects include “My future seems dark to me.“ The scale is divided into three dimensions: feeling of future, loss of motivation and future expectation. Respondents were asked to answer true (1) or false (2), with higher scores indicating greater thoughts and feelings of hopelessness [42]. In this study, the Cronbach’s α coefficients of this scale was 0.705.

#### The perceived stress scale (PSS)

This 10-item questionnaire [43] assessed how stressful individuals perceived different experiences over the past month. Each item used a five-point Likert scale to ask how often different feelings and thoughts (e.g., feeling upset about something unexpected) had occurred in the past month, with 0 being “never” and 4 being “often.“ The higher the score, the more stress you have felt in the past month. In this study, the Cronbach’s α coefficients of this scale was 0.968.

### Statistical analysis

All data analyses in this study were performed using SPSS 22.0. First, Harman’s one-factor analysis was used to check for common method biases. After principal component analysis, nine eigenvalues > 1 were extracted, and the first factor explained 26.11% of the variance (< 40%), demonstrating that there was no serious problem with common method biases. Second, descriptive statistics and correlations among variables were assessed. Third, both the mediation effect of hopelessness and the moderating role of psychological stress were tested by respectively using Model 4 and Model 7 of PROCESS macro for SPSS [44]. An effect was deemed significant when the 95% confidence interval (CI) does not include zero, based on a bootstrap random sample (n = 5000). All variables were standardized before entering the mediation model and demographic variables were used as covariates of the model.

## Results

### Descriptive statistics and correlation analysis

Means, standard deviations (SD), and Pearson correlation coefficients among main variables are presented in Table 1. Specifically, perceived social support was positively correlated to mental health, and negatively correlated with hopelessness. Hopelessness was negatively correlated with mental health, and there was a negative relationship between psychological pressure and mental health. Moreover, psychological pressure is not associated with hopelessness or perceived social support.

**Table 1:** Descriptive statistics and correlations of all variables (N = 672)

	M (SD)	1	2	3	4	5	6	7
1. Gender	–	1						
2. Age	20.43 ± 1.68	−0.097*	1					
3. Urban − rural provenance	–	0.236**	0.026	1				
4. Duration of sports careers	–	−0.236**	0.277**	−0.217**	1			
5. Perceived social support	60.77 ± 14.52	−0.056	−0.034	−0.128**	0.025	1		
6. Hopelessness	6.51 ± 3.31	−0.064	0.059	0.061	0.034	−0.383**	1	
7. Psychological pressure	23.59 ± 7.75	0.116**	−0.034	0.047	0.001	−0.024	−0.016	1
8. Mental health	4.00 ± 0.91	0.092*	−0.094*	−0.020	−0.051	0.385**	−0.457**	−0.191**

Note: *p < 0.05; ** p < 0.01

### Mediation analysis

Model 4 of the PROCESS was used to examine whether hopelessness mediated the link between perceived social support and mental health. After controlling for demographic variables, the results showed that (Table 2) perceived social support was negatively associated with hopelessness (β = −0.147, p < 0.001), which in turn was related to mental health (β = −0.097, p < 0.001). The residual direct effect was significant (β = 0.192, p < 0.05). Therefore, hopelessness partly mediated the relation between perceived social support and mental health (indirect effect = 0.102, Boot SE = 0.014, 95% CI = 0.075, 0.132). This model accounted for 34.7% of the variance in mental health, thus supporting Hypothesis 2.

**Table 2:** Testing the mediation effects of perceived social support on mental health (N = 672)

Independent variables	Model 1 (Mental health)		Model 2 (Hopelessness)		Model 3 (Mental health)	
	*β*	*t*	*β*	*t*	*β*	*t*
Gender	0.149	2.078*	−0.177	−2.293*	0.212	2.765**
Age	−0.033	−1.603	0.018	0.816	−0.039	−1.790
Urban − rural provenance	0.033	0.468	0.073	0.964	0.007	0.096
Duration of sports careers	−0.013	−0.260	0.032	0.601	−0.024	−0.457
Perceived social support	0.254	7.029***	−0.383	−10.665***	0.389	10.899***
Hopelessness	−0.353	−9.783***				
R²	0.521		0.396		0.408	
F	41.244***		24.818***		26.575***	

Note: *p < 0.05; *** p < 0.001

### Moderation analysis

Further, Model 7 of the PROCESS was used to test the moderated mediation hypothesis. After controlling for demographic variables, the results (see Table 3) indicated that perceived social support was positively associated with hopelessness (β = −1.115, p < 0.001), while psychological pressure was not related to hopelessness (β = − 0.007, p > 0.05) (Model 1). Moreover, the interaction of perceived social support and psychological pressure was positively correlated with hopelessness (β = −0.029, p < 0.001) (Model 1), indicating that psychological pressure moderated the relationship between perceived social support and hopelessness.

**Table 3:** Testing the moderated mediation effects of perceived social support on mental health of college athletes (N = 672)

Independent variables	Model 1 (Hopelessness)		Model 2 (Mental health)	
	*β*	*t*	*β*	*t*
Gender	−0.194	−2.502*	0.149	2.078*
Age	0.018	0.820	−0.033	−1.603
Urban − rural provenance	0.074	0.985	0.033	0.468
Duration of sports careers	0.027	0.516	−0.013	−0.260
Perceived social support	−0.408	−11.027***	0.254	7.029***
Psychological pressure	−0.017	−0.477		
Perceived social support × Psychological pressure	−0.081	−2.623***		
Hopelessness			−0.353	−9.783***
R²	0.407		0.521	
F	18.875***		41.244	

Note: **p < 0.01; ***p < 0.001

**Fig. 2:**
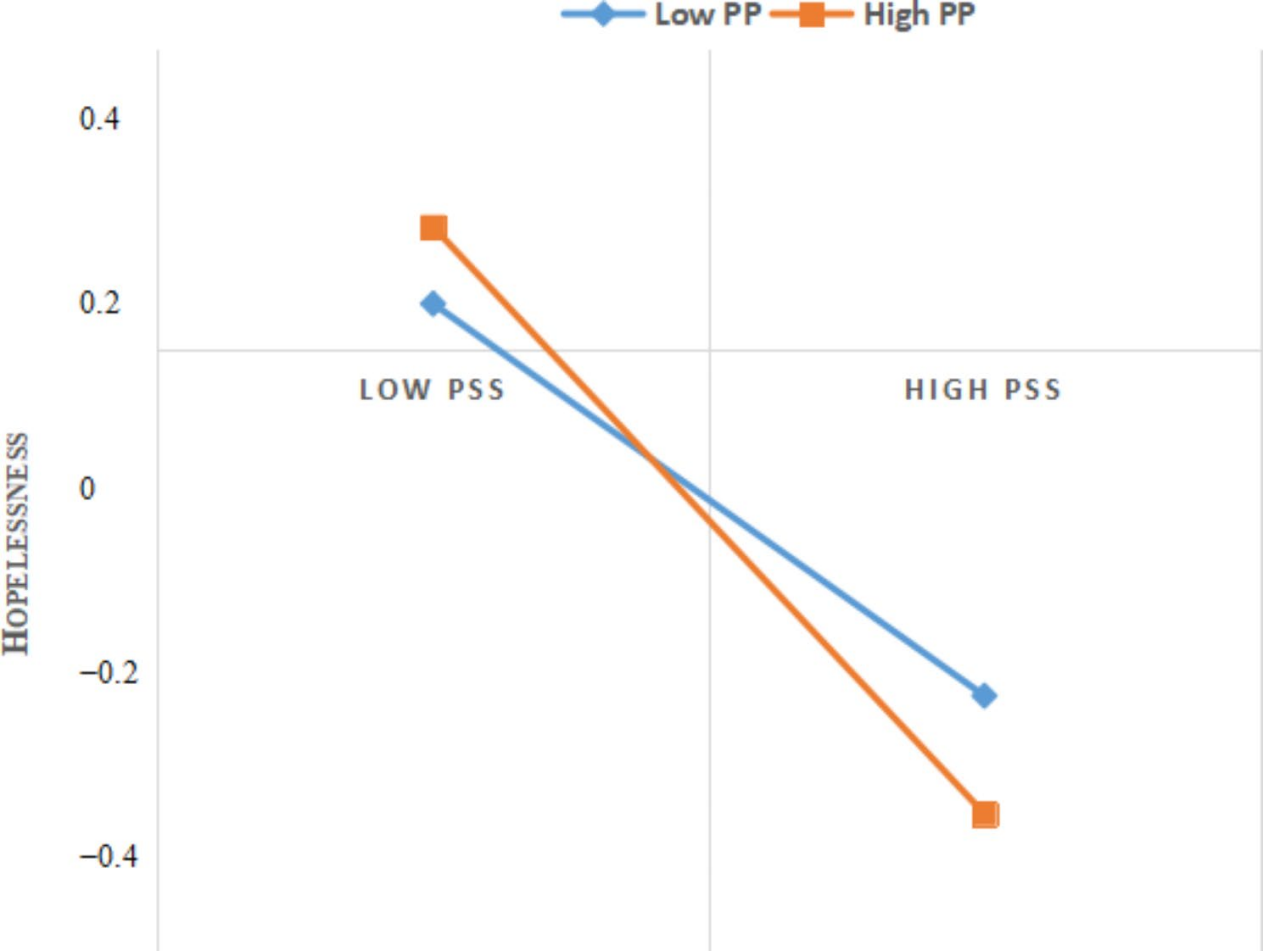
Interaction between perceived social support and psychological pressure on mental health. PSS: perceived social support; PP: psychological pressure

To facilitate the interpretation of the moderating effect of the psychological pressure, the plot of the link between perceived social support and mental health at two levels of psychological pressure (M + 1SD and M − 1SD) is presented in Fig. 2. Simple slope tests indicated that perceived social support was negatively correlated with hopelessness for both athletes with low − level psychological (M − 1SD) and those with high-level psychological pressure (M + 1SD). Bias corrected bootstrap analyses further showed that for athletes with low − level psychological pressure, the indirect effect between perceived social support and mental health was significant (β = −0.089, SE = 0.114, 95% CI = [− 1.118, − 0.669]. For athletes with high − level psychological pressure, the indirect effect between perceived social support and mental health was stronger (β = −1.337, SE = 0.147, 95% CI = [− 1.627, − 1.048]). With a formal test of moderated mediation, which assesses the index of moderated mediation and its corresponding CI, we find that the index equals 0.029, and its CI does not include 0 [0.009, 0.050]. Therefore, psychological pressure moderated the link between perceived social support and mental health, which supports Hypothesis 3.

## Discussion

The present study established a moderated mediation model by jointly considering the roles of hopelessness and psychological pressure. As expected, the results confirmed the mediating effect of hopelessness in the link between perceived social support and mental health among Chinese college football athletes during the COVID-19 pandemic, and this association was moderated by the psychological pressure.

The results of this study proved the positive association between perceived social support and mental health among Chinese college football athletes. Chinese college football athletes who perceived higher level of social support during the COVID-19 pandemic reported better mental health condition. The results of this study is consistent with previous studies and hypothesis 1 has been confirmed. Studies have shown that mental health during crisis events is often linked to social support [45]. In the context of the current COVID-19 pandemic, the government has imposed “physical distancing” to control infection, which may lead to individual isolation [46]. The potential consequences of social support on college football athletes’ mental health during epidemic outbreaks, however, have received little prior research. Studies during the COVID-19 pandemic have shown that low levels of social support are associated with poor mental health outcomes among Chinese college students [47] or Chinese adolescents [48], moreover, high-level perceived social support is associated with a reduced risk of poor mental health outcomes such as suicidal ideation [49]. Physical distancing as a way to avoid spreading the virus, however, may reduce social interactions that keep people mentally healthy. Research also confirmed that perceived social support can affect people’s behavior through subjective perceived psychological reality, and it is more likely to show beneficial functions for their mental health [50]. As an important external resource, social support can significantly improve the investment level of college athletes [51], which has a positive prediction effect on sports performance and can also help relieve the psychological pressure of athletes. During the COVID-19 pandemic, college athletes have been in a closed and high-intensity training environment for a long time. They not only have to bear various external pressures (such as external expectations, harsh criticism from coaches), but also have to deal with their own inner confusion and confusion. As a kind of stress buffer, perceived social support can make college athletes have confidence and ability to deal with various pressures and improve their mental health level.

Consistent with our expectations, this study found that hopelessness mediated the link between perceived social support and mental health. This result corresponds with previously reported conclusions [26, 52, 53]. Stressful events have been reported to affect human mental/morale levels, leading to hopelessness [54]. The explanation is that COVID-19, as a major negative stress event, exerts a strong psychological impact on individuals. College football players are faced with the double pressure of study and competition, and their inner resources are consumed greatly. College athletes with higher understanding of social support are more likely to use social support to self-regulate and solve problems in the face of epidemic stress, so as to alleviate the adverse effects of epidemic stress and reduce their hopelessness level. If college athletes believe that they lack the support of society, school or family, they may think that they have no hope to change their situation, improve their quality of life and happiness, and feel hopeless [55]. Hopelessness is the product of a key belief that the future will produce bad outcomes that people cannot influence. In addition, Our research also support Rudd’s theory, which placed perceived social support as a cognitive-emotional factor parallel to hopelessness [56]. The hopelessness of college athletes not only causes and aggravates a series of negative emotions, but also promotes negative behaviors, and seriously affects their mental health and quality of life. However, our research has found that psychological pressure is not significantly related to perceived social support and hopelessness, which is not consistent with previous studies [34, 57]. The possible reason is that there may be important mediating variables between the three, rather than a linear relationship. In addition, special group characteristics or cultural differences may be important reasons for this result.

In addition to the mediating role of hopelessness, another important contribution of these findings is that psychological pressure can moderate the indirect link between perceived social support and hopelessness. Specifically, psychological pressure seems to have a negative psychological effect only on college athletes who perceive low levels of social support, but less on college students who experience high levels of social support, which is related to the decrease in hopelessness. This means that the negative function of psychological pressure seems to diminish as the level of perceived social support increases. Social support is considered to be the most powerful coping strategy, providing individuals with the ability to successfully cope with stressful conditions and tolerate problems. When faced with high life stress, people with less social support were more likely to experience higher levels of hopelessness [26]. The conservation of resource model [58] indicates that: (1) individuals will consume resources when dealing with stress; (2) Individuals who lack resources are not only vulnerable to the pressure brought by resource loss, but this pressure will lead to the investment of other resources exceeding their income, thus accelerating the loss of resources; 3) The resources owned by individuals are closely related to adaptation, and the less resources they have, the worse their adaptation. Combined with the concept of perceived social support [10], it can be seen that perceived social support itself is a kind of social psychological resources, and lack of such resources may lead to stress and serious mental health problems. Perceived social support can enable college athletes to maintain a more positive attitude towards life’s problems and increase their confidence in dealing with them. The COVID-19 pandemic situations may deplete athletes’ internal resources and enable them to assess their social support, in which case people with low perceived levels of social support may focus more on their weak support, leading to increased hopelessness [26]. Our study also found that for college athletes with high psychological stress, as the level of social support increased, their sense of hopelessness also decreased significantly. However, our study showed that college athletes with low psychological stress score higher on hopelessness than those with high psychological stress in the case of high perceived social support. The reason may be that: for individuals with low levels of psychological stress, the understanding of social support can provide individuals with a wide range of psychological, emotional and material resources, improve their ability to cope with pressure, and thus reduce the hopelessness; however, for individuals with a high level of psychological stress, according to the stress process model [59], social support will help to reduce the pain of people under high pressure. Therefore, compared with individuals with a low level of stress, individuals with high perceived social support may tend to turn stress into motivation, reduce feelings of hopelessness and have higher positive feelings [60]. Considering that, college athletes with higher perceived social support are more likely to use social support to self-regulate and solve problems when facing the stress of epidemic events, and the adverse effects brought by stress, thus reducing their hopelessness level.

The study’s findings have three important theoretical implications. First, the research was designed to investigate college football players who are in the midst of this particular phase of the COVID-19 pandemic, and to expand the research into social support insights to a broader population. Previous research has focused on college students’ perceived social support, such as understanding the effects of social support on college students’ physical activity behavior, happiness and health risk behavior [61–63], with little focus on college athletes. Second, to the best of our knowledge, this study explores for the first time the role of hopelessness in understanding the relationship between perceived social support and mental health, as well as the protective role of psychological pressure and hopelessness, thereby enriching and expanding relevant research on mental health. Thirdly, this study constructs a moderating mediation model, focusing on the mediating effect of hopelessness and the moderating effect of psychological pressure. This model can provide important empirical evidence for understanding the mechanism of understanding the impact of increased social support on the mental health of college football athletes during the COVID-19 pandemic. In addition, the findings have important practical implications. Mental health has received widespread attention as the COVID-19 pandemic has progressed. From the perspective of positive psychology, this study provides important significance for the development of intervention strategies to improve the mental health of college athletes, especially the new idea of promoting mental health by reducing the sense of hopelessness and psychological stress. The results of this study are expected to have a significant impact on future practice when determining how to protect college athletes from the negative effects of the pandemic on their mental health. The government and people from all walks of life provide the corresponding social support to the public. University athletes can prevent the negative psychological effects of depression, stress and other reactions produced by the novel coronavirus by feeling and understanding the actual social support they receive. The more social support they can perceive, the more they can adjust and enhance their physical and mental health. Alleviating the negative emotions of coping with sudden public health events has a positive impact on their physical and mental health.

Notwithstanding the above, a number of limitations should be noted. First of all, data collection is based on self-reported surveys, which may affect the validity of conclusions. Future studies should adopt multiple methods to test the stability of results. Second, due to the use of cross-sectional data, causal conclusions cannot be drawn in this study. In future studies, causal relationships between these key variables should be determined by longitudinal or experimental design. Third, the subjects of this study are all from mainland China, which may limit the generality of the results. Therefore, it is necessary to test the conclusions of this study in different cultural contexts in future studies. Fourth, this study believes that hopelessness is an internal mechanism of perceived social support and mental health, and other possible mechanisms should be considered more in future studies. Moreover, future studies can apply multi-level modeling.

## Conclusion

The COVID-19 pandemic is not ended yet and is depleting human psychological resources. Measures taken by governments and schools to reduce hopelessness among college athletes during and after COVID-19 are critical to maintaining good mental health. In summary, the present study contributes to the literature by providing a unique perspective for understanding how and when perceived social support is associated with mental health of Chinese college football athletes. The results showed that hopelessness mediated the link between perceived social support and mental health. Moreover, a low-level psychological pressure can act as a buffer for athletes who experienced less perceived social support. At the same time, reducing the psychological pressure they may experience may help enhance the perception of social support in difficult circumstance, and may also help enhance the ability of college athletes to cope with adverse emotions and maintain mental health.

## Data Availability

Data is available on request from the corresponding author.
